# Beyond the matrix: structural and physiological advancements in mitochondrial calcium signaling

**DOI:** 10.1042/BST20220317

**Published:** 2023-03-24

**Authors:** Melissa J.S. MacEwen, Yasemin Sancak

**Affiliations:** Department of Pharmacology, University of Washington, Seattle, WA 98195, U.S.A.

**Keywords:** calcium signaling, mitochondria, mitochondrial calcium uniporter, mitochondrial signaling

## Abstract

Mitochondrial calcium (Ca^2+^) signaling has long been known to regulate diverse cellular functions, ranging from ATP production via oxidative phosphorylation, to cytoplasmic Ca^2+^ signaling to apoptosis. Central to mitochondrial Ca^2+^ signaling is the mitochondrial Ca^2+^ uniporter complex (MCUC) which enables Ca^2+^ flux from the cytosol into the mitochondrial matrix. Several pivotal discoveries over the past 15 years have clarified the identity of the proteins comprising MCUC. Here, we provide an overview of the literature on mitochondrial Ca^2+^ biology and highlight recent findings on the high-resolution structure, dynamic regulation, and new functions of MCUC, with an emphasis on publications from the last five years. We discuss the importance of these findings for human health and the therapeutic potential of targeting mitochondrial Ca^2+^ signaling.

## Introduction

Mitochondrial calcium (Ca^2+^) uptake was first observed as an *in vitro* phenomenon in the early 1960s [1,2]. It was quickly recognized as an important regulator of mitochondrial bioenergetics through Ca^2+^-mediated activation of the TCA cycle. This foundational work paved the way for decades of research on the functions of mitochondrial Ca^2+^ uptake in physiology and diseases. Yet, the molecular identity of the uniporter remained a mystery for decades. The proteins responsible for mitochondrial Ca^2+^ influx into the mitochondrial matrix — the mitochondrial calcium uniporter (MCU) and its regulatory proteins — were identified over the last 13 years. These discoveries dramatically accelerated efforts to understand the regulation and function of mitochondrial Ca^2+^ uptake. Ca^2+^ influx across the inner mitochondrial membrane (IMM) is now recognized to govern numerous aspects of biology, ranging from ATP production via oxidative phosphorylation, to cytoplasmic Ca^2+^ signaling in a variety of tissues [3–6], to regulation of immunological synapses [7]. High mitochondrial matrix [Ca^2+^] can also lead to opening of the mitochondrial permeability transition pore (mPTP), a structure that is responsible for rapid release of Ca^2+^ and other small molecules from the mitochondrial matrix to the cytosol, which can cause cell death [8]. Mitochondrial Ca^2+^ signaling has also been found to play a significant role in human diseases including neurological disorders such as amyotrophic lateral sclerosis, Friedreich's ataxia, Charcot–Marie–Tooth disease [9], Alzheimer's disease [10], as well as heart failure [11] and lysosomal storage disorders [12].

The field of mitochondrial Ca^2+^ biology continues to advance rapidly. Here, we review recent publications centered on the MCU, with an emphasis on its structure, novel regulatory mechanisms, and its emerging roles in development, mitochondrial diseases and immunity. We contextualize these findings in the broader field, while emphasizing publications from the past five years. Many excellent reviews concerning the biology of mitochondrial Ca^2+^ signaling were published within this same time frame. Of note are those concerning the mechanisms of mitochondrial Ca^2+^ signaling in health and disease, as well as those exploring the role of mitochondrial Ca^2+^ signaling in cardiac disease, diabetes, cellular senescence, cancer, and neurodegeneration. We encourage the reader to pursue these reviews to gain an even deeper understanding of this rich field [13–19].

## Mechanisms of mitochondrial Ca^2+^ influx, efflux, and sequestration

**Figure 1.**
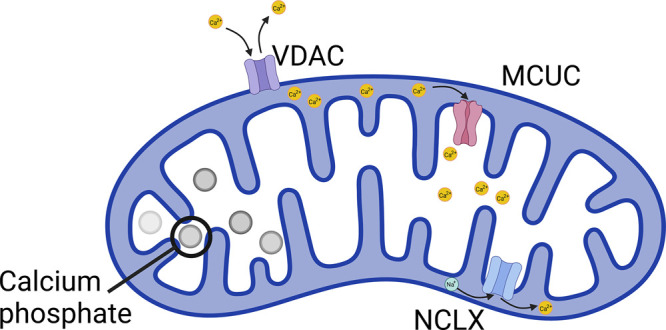
Mechanisms of mitochondrial Ca^2+^ influx, efflux, and sequestration. Mitochondria are organelles with an outer mitochondrial membrane (OMM) and inner mitochondrial membrane (IMM). Ca^2+^ ions likely diffuse through the OMM via VDAC proteins. Bulk transport of Ca^2+^ across the IMM and into the mitochondrial matrix requires the mitochondrial calcium uniporter complex (MCUC), a highly selective ion channel. NCLX, a Ca^2+^/Na^+^ exchanger, enables the majority of Ca^2+^ efflux from the mitochondria. Calcium phosphate deposits within the mitochondria sequester Ca^2+^ and may serve as Ca^2+^ reservoirs that can be mobilized if needed.

To maintain homeostasis, mitochondria must precisely balance the influx, sequestration, and release of Ca^2+^ into and from the mitochondrial matrix. Characterizing the machinery and chemistry central to these processes has clarified the regulation and biological implications of mitochondrial Ca^2+^ cycling and signaling. The main players of mitochondrial Ca^2+^ homeostasis are shown in Figure 1 and described in more detail below.

### Mechanisms of influx

Mitochondrial Ca^2+^ uptake from the cytosol to the mitochondrial matrix faces two primary physical barriers: the outer mitochondrial membrane (OMM) and the IMM. Ca^2+^ transport across the OMM remains poorly understood as the OMM is not believed to have a specific Ca^2+^ transporter [20]. Rather, the voltage-dependent anion channel (VDAC) is believed to passively permit the diffusion of metabolites and solutes such as Ca^2+^. VDAC was recently demonstrated to form multi-protein complexes with the Ca^2+^ channels of other organelles, thus facilitating highly efficient Ca^2+^ transfer through the OMM [21].

Once past the OMM, the vast majority of Ca^2+^ travels from the intermembrane space (IMS) to the mitochondrial matrix via rapid bulk entry through the MCU. MCU is a pore-forming protein that resides in the IMM and is the eponymous protein of the mitochondrial calcium uniporter complex (MCUC). MCU-facilitated Ca^2+^ flux is dependent on mitochondrial membrane potential. The uniporter is a Ca^2+^ selective channel, and patch-clamp experiments of the IMM reveal that it has a Ca^2+^ affinity of 2 nM or less [22].

**Figure 2.**
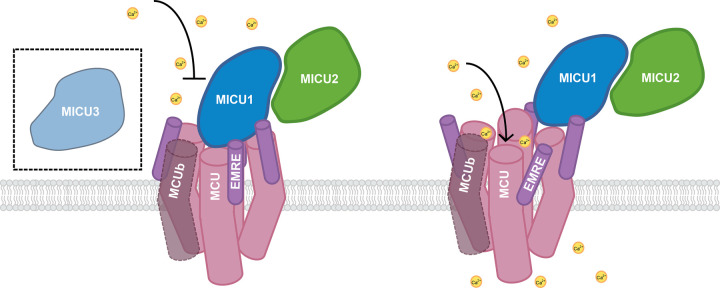
The mitochondrial calcium uniporter complex. The mitochondrial calcium uniporter complex (MCUC) is composed of membrane proteins MCU, MCUb, EMRE and IMS-localized proteins MICU1 and MICU2. MCU is the pore-forming protein and requires EMRE to form a functional channel in multicellular and some unicellular organisms. The incorporation of MCUb into the MCUC modulates mitochondrial Ca^2+^ flux and prevents mitochondrial Ca^2+^ overload. The MICU proteins — MICU1, MICU2, and MICU3 — block the entrance of Ca^2+^ into the pore, except during Ca^2+^ signaling events. During signaling events, the MICU proteins dissociate from the MCUC pore, remaining tethered via MICU1–EMRE binding. MICU1 forms a disulfide-bonded heterodimer with either MICU2 or MICU3, though MICU3 has only been identified in brain tissue. Arrows indicate the presence or absence of Ca^2+^ transport, with MCUC illustrated in a closed, non-conductive conformation (left) or an open, Ca^2+^-conducting conformation (right).

Additional core MCUC proteins include Mitochondrial Calcium Uptake (MICU) homologs MICU1–3, Essential MCU Regulator (EMRE), and MCUb. The MICU proteins play distinct, crucial roles in setting the threshold for uniporter Ca^2+^ uptake and its potentiation. They also contribute to specific Ca^2+^ regulation of the channel through their EF-hand Ca^2+^-binding domains [23–30]. The consensus of the field is that, when the cytosolic [Ca^2+^] is low, MICU1 blocks the MCU pore to prevent mitochondrial Ca^2+^ overload [23,27,28,31]. When IMS [Ca^2+^] increases, Ca^2+^ binds to the EF-hands of the MICU proteins. MICU1 — which forms a disulfide-bonded heterodimer with either MICU2 or MICU3 — then dissociates from MCU, enabling Ca^2+^ conductance. Recent structural work supports this consensus, as discussed in detail below. EMRE, a small transmembrane protein, is required for both MCU–MICU1 interaction and Ca^2+^ conductance through MCU [32–34]. MCUb is a paralog of MCU, but it does not form a functional Ca^2+^ channel and seems to have a negative regulatory role in the MCUC. MICU3, a MICU1 paralog, is mostly found in brain tissue and enhances Ca^2+^ uptake in neuronal mitochondria [35] (Figure 2).

Structural and biochemical studies have shown that MCU and its associated proteins form the MCUC, a large holocomplex whose structure and interactions are discussed in detail below. Two other IMM proteins, Mitochondrial Calcium Uniporter Regulator 1 (MCUR1) and Solute Carrier 25A23, also regulate the activity of the uniporter despite not being a part of the core complex [36,37]. MCUR1 is a scaffold protein that binds to MCU and EMRE and is required for MCUC assembly. SLC25A23 augments mitochondrial Ca^2+^ uptake, however the exact mechanism of this regulation remains elusive.

### Mechanisms of Ca^2+^ efflux and sequestration

Matrix Ca^2+^ is maintained at a far lower resting concentration than the concentration reached after a Ca^2+^ signaling event [38]. This regulation is crucial; excessive mitochondrial Ca^2+^ accumulation can be hazardous, as it can lead to mitochondrial Ca^2+^ overload or opening of the permeability transition pore [39]. To restore resting Ca^2+^ levels, exchangers and antiporters facilitate the exit of Ca^2+^ from mitochondria. The Na^+^/Ca^2+^ exchanger NCLX is the dominant Ca^2+^ efflux pathway [40]. NCLX is expressed on the IMM of many tissues, in an expression pattern similar to that of MCU [41,42]. Ca^2+^ ions are also stored as calcium phosphate deposits in the mitochondrial matrix. Fast sequestration of Ca^2+^ in the form of calcium phosphate after mitochondrial Ca^2+^ entry is thought to play an important role in reducing the concentration of free matrix [Ca^2+^]. These deposits are also thought to serve as Ca^2+^ reservoirs that can be mobilized if needed. Although the function of mitochondrial calcium phosphate deposits is still under debate, their visualization using cryo-scanning transmission electron tomography shows that these deposits form independently of MCU, disappear if mitochondrial membrane potential is lost, and do not preferentially form in mitochondria in a particular cellular location [43].

## High-resolution uniporter structures unveil new regulators and mechanisms

A remarkable series of studies have recently shed light on the structures of the individual proteins of the MCUC, as well as the uniporter holocomplex in its Ca^2+^-bound and Ca^2+^-free states [30,44–50]. By defining specific interactions of the uniporter proteins and identifying a lipid molecule that interacts with the uniporter channel, these publications have helped to develop a more refined model of uniporter regulation and the functions of its individual subunits.

In the wake of these publications and their predecessors, the following is clear: MCU is a transmembrane protein that spans the IMM and contains two transmembrane-spanning helices, TM1 and TM2. TM2 of MCU forms the protein's Ca^2+^-conducting pore. Although MCU was known to oligomerize prior to 2018 [32,51], cryo-EM structures established the presence of a ‘dimer of dimer' structure composed of four MCU protomers [44–47], with different symmetric arrangements of the soluble and transmembrane domains [47]. There are four EMRE subunits per functional channel. Structurally, EMRE participates in the formation of functional channels by interacting with MCU at the matrix channel opening. This interaction stabilizes a region at the inner leaflet of the IMM, which is otherwise flexible. This region is proposed to occlude the matrix side of the Ca^2+^ pore, preventing escape of Ca^2+^ ions into the matrix and regulating channel gating through binding-induced structural changes in the channel [50,52]. Related work from our laboratory independently validated several of these structural findings biochemically, by using a suite of protein chimeras composed of domains from *H. sapiens* MCU (HsMCU) and *D. discoideum* MCU (DdMCU) [53]. *D. discoideum* MCU is functional in the absence of EMRE, which enabled us to characterize the aspects of HsMCU that make it dependent on EMRE for Ca^2+^ conductance. This work led to the identification of a 10-amino acid region in HsMCU that renders it EMRE-dependent; we termed this region EMRE dependence domain (EDD). This biochemically identified EDD region overlaps with a 6-amino acid stretch first identified by Wang *et al.* [50] in a mammalian EMRE–MCU structure that they show is important for EMRE function.

A second function of EMRE is facilitating the interaction of MICU1 with MCU through EMRE's positively charged DDD domain that faces the IMS. Using structural data from the MICU1–MICU2 heterodimer and *in vitro* binding experiments, Wu *et al.* [49] showed that the DDD domain interacts with a Ca^2+^-induced alkaline stretch in MICU1. This Ca^2+^-enhanced interaction between EMRE and MICU1 is thought to prevent complete dissociation of MICU1 from the complex in the presence of Ca^2+^. Two publications also suggested another important function of the EMRE–MICU1 interaction: potentiation of uniporter activity [30,54]. The authors propose that, by binding to EMRE when Ca^2+^ is present, MICU1 alters MCU–EMRE interaction, which results in increased uniporter conductance.

## Controlling MCUC composition and stability to tune mitochondrial Ca^2+^ uptake

The activity of the uniporter varies across physiological conditions and cell types [55]. Recent work highlighted the importance of two primary modes of fine-tuning uniporter activity: steady state differences in the expression of MCUC proteins across tissues; and regulation of MCUC proteins in response to stress, disease, or other alterations to cellular or organismal physiology.

MCU and MICU1 are posttranslationally modified in response to various stimuli, which lead to changes in uniporter activity and have been reviewed before [56]. Here, we focus on new research indicating that changes to uniporter subunit composition or altered stability of the complex is a novel mode of uniporter regulation. Such changes often happen at the level of individual complex proteins, and can have dramatic, long-lasting effects on cellular and tissue wide Ca^2+^ signaling and metabolism. For example, during chronic stress, increased MCUb incorporation into the complex limits mitochondrial Ca^2+^ overload and counteracts mitochondrial damage, as seen in mice in response to cardiac injury [57]. Consistent with this observation, Huo *et al.* [58] demonstrated that cardiomyocyte-specific MCUb knockout mice exhibited increased pathological cardiac remodeling and infarct expansion following ischemic injury. This same group found the inverse in cardiomyocyte specific MCUb overexpressing mice, highlighting the importance of proper uniporter composition for homeostasis.

A similar mechanism of altered uniporter composition is observed in failing human heart tissue. The relative mRNA abundance of MICU1 to MCU is tissue-specific and is particularly low in cardiac muscle. During heart failure, MICU1/2 expression increases, while MCU expression stays the same. This altered composition likely changes the gating and activity of the uniporter in failing human hearts and may contribute to decreased cardiac contractile function in heart failure [59]. MICU3 is another uniporter regulatory protein whose presence in the complex substantially alters the physiological performance of specific tissues. MICU3 is a paralog of MICU1 and MICU2. It is mostly found in the brain and binds specifically to MICU1 to enhance Ca^2+^ uptake [35]. If MICU3 is silenced in primary cortical neurons, the Ca^2+^ signals spurred by synaptic activity are impaired. Finally, increased MICU1 expression and ensuing changes in Ca^2+^ signaling and metabolism are important for fibroblast to myofibroblast differentiation [60].

The precise regulation of EMRE has important consequences for human health. Under normal physiological conditions, EMRE that does not complex with MCU is rapidly degraded by the m-AAA protease [61]. This process is essential to maintaining the balance of MCU–EMRE that are assembled with an adequate ratio of MICU1/MICU2 ‘gatekeeper’ subunits [62]. Mutations in mitochondrial m-AAA proteases, which are linked to neurodegeneration in spinocerebellar ataxia (SCA28) and hereditary spastic paraplegia (HSP7), lead to accumulation of EMRE and formation of excess MCU–EMRE complexes that are not gated by MICUs. This eventually leads to unregulated mitochondrial Ca^2+^ entry and overload. This has been proposed to contribute to neurodegeneration in these debilitating diseases.

The stability of MCU is also carefully regulated, as recently shown by two elegant studies. In 2020, using functional experiments in a yeast heterologous uniporter expression system, Ghosh *et al.* [63] showed that cardiolipin plays an essential role in stabilizing MCU. This finding may clarify the pathologically low cardiolipin and uniporter levels observed in patients with Barth syndrome, a disease characterized by partial loss of cardiolipin. Furthermore, Dr. Chaudhuri and coworkers discovered a novel link between OXPHOS function and MCU. In healthy cells, reactive oxygen species that leak from Complex I of the electron transport chain damage MCU and lead to its degradation. In Complex I deficiency, MCU is stabilized, and the number of functional uniporter channels increases, leading to an increase in overall MCUC activity. Most importantly, the authors showed that uniporter function prolongs survival of OXPHOS-deficient mice and *Drosophila*, thereby identifying a previously unknown function for the uniporter [64].

## Expanding the functions of mitochondrial Ca2^+^ flux

Targeted perturbation of mitochondrial Ca^2+^ flux in animal models and phenotypes of patients with uniporter gene mutations highlight the importance of this pathway in development, immunity, neuromuscular system and metabolic health. Likely due to highly tissue-specific nature of Ca^2+^ signaling and mitochondrial functions, uniporter activity has been shown to regulate distinct pathways across different tissue types. Despite this functional diversity, altered uniporter regulation fundamentally produces a phenotype through either altered metabolic regulation, and/or altered cellular Ca^2+^ signaling. Here, we non-exhaustively highlight recent findings on the physiological roles of MCU.

### Emerging roles of the uniporter in early development and differentiation

The first report of MCU knockout (KO) mice was somewhat of a surprise for mitochondrial community: loss of MCU was compatible with life, but only on a mixed background [65]. Since then, the same phenotype of viability on a mixed background has been shown for EMRE KO mice [66]. MCU loss is also tolerated in the fly and the worm [55,67]. Despite being viable, loss of MCU causes reduced viability in the mouse. The KO mice are observed at much lower ratios than expected from a heterozygous mating [68]. This suggests the presence of modifiers (genetic or environmental) that allow survival of animals past a certain developmental stage in the absence of mitochondrial Ca^2+^ uptake. Important roles for mitochondrial Ca^2+^ uptake in early development were recently shown in different model organisms and systems. In *Xenopus* eggs, early cell divisions after fertilization require ROS generated by the mitochondria, which is fueled by MCU-mediated mitochondrial Ca^2+^ uptake [69]. Modulation of MCU activity is also observed in human embryonic stem cells (hESCs): repression of MICU1 by Foxd1 is essential for proper differentiation of hESCs into induced pluripotent stem cells [70] (hiPSCs). The authors in this study conclude that the presence of MICU1 modulates periodic cytosolic Ca^2+^ oscillations necessary for differentiation. These new roles of the uniporter in early development help explain the viability (albeit low) of MCU KO animals on a mixed background. Nevertheless, how MCU KO animals survive, and what type of compensatory changes take place in these survivors, are still unknown.

Although no MCU mutation has ever been identified in humans, a recent preprint by Bulthuis *et al.* [71] reported two patients with EMRE mutations that lead to a loss of EMRE protein. Muscle breakdown was a common phenotype observed in both patients, who otherwise showed diverse symptoms. In addition, several patients with MICU1 mutations have been reported to date (OMIM #615673), most of which present with neuromuscular problems. A neuron-specific MICU1 KO mouse model recapitulates the phenotypes observed in patients and whole body MICU1 KO mice, suggesting that neuronal abnormalities are the main cause of the observed phenotypes in patients [72]. Nevertheless, phenotypic diversity observed in humans again underlines the importance of other modifiers and compensatory mechanisms in mitochondrial Ca^2+^ regulation. For example, the appearance and bodyweight of MICU1 KO mice become more similar to wildtype mice over time due to down-regulation of EMRE expression [27].

### Emerging tissue-specific roles of the uniporter

Several recent studies highlight the diverse, tissue-specific functions of the uniporter. They also point to a need for further research on uniporter regulation and function, as several contradictory phenotypes associated with the uniporter have been reported. For example, in a 2020 publication, Flicker *et al.* [73] generated a brown adipose tissue (BAT)-specific MCU KO mouse and reported that MCU is not required for brown fat energetics. In contrast, Xue *et al.* [74] found that in the BAT, the uniporter function is important for thermogenesis in response to cold. The authors suggested that these disparities are due to the differences in the genetic background of the mice used in these two papers, but they could also stem from experimental differences such as fasting before cold exposure in the second study. In addition, several papers reported contradicting phenotypes associated with loss of uniporter function in the heart. As proposed by Garbincius *et al.* [75], some of these differences can be attributed to cellular adaptations in response to chronic loss of uniporter function but are absent from its acute inhibition.

The importance of metabolism, mitochondria and cellular Ca^2+^ signaling for proper functioning of the innate immunity is well appreciated [76]. The uniporter sits at the intersection of these regulators, and several papers showed important roles of the uniporter in different aspects of cellular responses to pathogens. In neutrophils, activation of mitochondrial Ca^2+^ uptake stimulates cell polarization and chemotaxis, which are required for effective removal of pathogenic species [77]. In addition, increased uniporter function is associated with phagocytosis-induced activation of NLRP3 inflammasome in macrophages [78]. Conversely, reduced uniporter activity due to MCUb expression decreases inflammation in macrophages [79]. Moreover, in epithelial cells that express pathogenic cystic fibrosis receptor, bacterial infection activates NLRP3 in an MCU-dependent manner [80]. Even though MCU activation is associated with worse cellular outcomes and more pathogen survival in the literature so far, whether modulating its activity pharmacologically would be beneficial or damaging to the cells is likely to depend on many factors at play, such as the duration of infection and detrimental effects of prolonged inflammation.

## Concluding remarks

Identification of the MCUC protein components accelerated research on numerous aspects of uniporter biology and led to exciting discoveries on the structure, regulation, and function of the uniporter, as well as its roles in diseases. Nevertheless, several fundamental questions about mitochondrial Ca^2+^ signaling and MCUC regulation at the level of transcription, post-transcription, translation, and posttranslation remain unanswered. For example, the presence of yet-uncharacterized, uniporter-independent mitochondrial Ca^2+^-entry pathways have been reported [81,82]. Identification of these pathways could alter our understanding of how mitochondrial Ca^2+^ signaling reflects and responds to mitochondrial and cellular needs. Furthermore, upstream signals that change MCUC protein transcription or the generation of alternative splice variants remain to be characterized and may help explain tissue-specific differences in uniporter function [83].

Finally, while mitochondrial Ca^2+^ signaling is generally considered necessary for proper mitochondrial and cellular health, the viability of MCU KO cells in mixed background mice suggests that the loss of uniporter function may be tolerated in normal tissues and may even have protective effects in the context of Ca^2+^-induced mitochondrial damage, as observed in hereditary ataxias. Though the therapeutic potential of MCUC inhibition is made uncertain by the diverse roles of mitochondrial Ca^2+^ flux in different tissues, and the cellular adaptations to altered uniporter function, the MCUC remains a promising therapeutic target. The field of mitochondrial Ca^2+^ signaling continues to develop rapidly and deepen our understanding of the mitochondria's role in health and disease. We look forward to what is to come.

## Abbreviations

BAT: brown adipose tissue
EDD: EMRE dependence domain
EMRE: essential MCU regulator
HsMCU: *H. sapiens* MCU
IMM: inner mitochondrial membrane
IMS: intermembrane space
KO: knockout
MCU: mitochondrial calcium uniporter
MCUC: mitochondrial calcium uniporter complex
MCUR1: mitochondrial calcium uniporter regulator 1
MICU: mitochondrial calcium uptake
OMM: outer mitochondrial membrane
VDAC: voltage-dependent anion channel
